# The stability analysis of a nonlinear mathematical model for typhoid fever disease

**DOI:** 10.1038/s41598-023-42244-5

**Published:** 2023-09-15

**Authors:** Ihsan Ullah Khan, Shahbaz Mustafa, Ali Shokri, Shuo Li, Ali Akgül, Abdul Bariq

**Affiliations:** 1 Department of Mathematics, Institute of Numerical Sciences, Gomal University, Dera Ismail Khan, 29050 KPK Pakistan; 2 Department of Mathematics, Faculty of Science, University of Maragheh, Maragheh, 83111-55181 Iran; 3 School of Mathematics and Data Sciences, Changji University, Changji, 831100 Xinjiang People’s Republic of China; 4 Department of Computer Science and Mathematics, Lebanese American University, Beirut, 5053 Lebanon; 5 Mathematics Research Center, Department of Mathematics, Near East University, Near East Boulevard, 99138 Nicosia, Mersin, Turkey; 6 Department of Mathematics, Art and Science Faculty, Siirt University, 56100 Siirt, Turkey; 7 Department of Mathematics, Laghman University, Mehtarlam City, Laghman 2701 Afghanistan

**Keywords:** Applied mathematics, Bacterial infection

## Abstract

Typhoid fever is a contagious disease that is generally caused by bacteria known as Salmonella typhi. This disease spreads through manure contamination of food or water and infects unprotected people. In this work, our focus is to numerically examine the dynamical behavior of a typhoid fever nonlinear mathematical model. To achieve our objective, we utilize a conditionally stable Runge–Kutta scheme of order 4 (RK-4) and an unconditionally stable non-standard finite difference (NSFD) scheme to better understand the dynamical behavior of the continuous model. The primary advantage of using the NSFD scheme to solve differential equations is its capacity to discretize the continuous model while upholding crucial dynamical properties like the solutions convergence to equilibria and its positivity for all finite step sizes. Additionally, the NSFD scheme does not only address the deficiencies of the RK-4 scheme, but also provides results that are consistent with the continuous system's solutions. Our numerical results demonstrate that RK-4 scheme is dynamically reliable only for lower step size and, consequently cannot exactly retain the important features of the original continuous model. The NSFD scheme, on the other hand, is a strong and efficient method that presents an accurate portrayal of the original model. The purpose of developing the NSFD scheme for differential equations is to make sure that it is dynamically consistent, which means to discretize the continuous model while keeping significant dynamical properties including the convergence of equilibria and positivity of solutions for all step sizes. The numerical simulation also indicates that all the dynamical characteristics of the continuous model are conserved by discrete NSFD scheme. The theoretical and numerical results in the current work can be engaged as a useful tool for tracking the occurrence of typhoid fever disease.

## Introduction

Infectious diseases can be transmitted among people either directly or indirectly. These diseases take place when germs enter into the body, enhance in quantity, and then cause a reaction of the body. Typhoid fever is a severe infection disease that can spread in the whole body, influencing numerous organs. If not treated on time, it can cause genuine difficulties and can be life threatening. It is caused by bacteria named Salmonella Typhi^1^. This bacteria is found in constipated food or water, and it causes illness when it enters the body through drinking or eating. Although, the sanitation of water coverage is improved but the spread of typhoid disease is still a noteworthy public health issue in several emergent countries^2^. The most common typhoid symptoms are migraine, stomach ache, knee pain, spine, muscular pain, lack of appetite, spew, dysentery, spots, and fever^3^.

Typhoid fever can become more dangerous if not treated instantly. It can damage the internal flow of blood and cause infection in the tissue that lies in the stomach^4^. Typhoid fever can be diagnosed by using some simple blood or stool tests. These tests identify the existence of Salmonella typhi in blood or stool samples. Typhoid fever preventive and control strategies include antibiotic treatments, stool standard precautions, vaccination, environmental sanitation and clean water^5^. Typhoid fever maybe treated with medicines and the symptoms improve within four weeks. The symptoms may return if treatment is not completed^6^. Over 110 years ago, the first typhoid vaccine was developed. Typhoid fever vaccines are available in two forms: oral and injectable. Vi polysaccharide vaccine is a licensed injectable vaccine which is secure and 65% defensive while Ty21a is a licensed liquid oral vaccine that can be used for children of 2 years and older. The oral vaccine is more costly than the Vi polysaccharide vaccine^7^. Many studies have been carried out over the last few decades, and different studies have resulted into different mathematical models^4–7^.

Numerous physical phenomena are being explained by researchers using fractional and integer order mathematical models^8–15^. The implementation of mathematical modelling enables us to focus on the process by which an infectious disease spreads throughout a region. To comprehend various infectious diseases and their dynamical properties, experts from all around the world developed several mathematical models^16–18^. Mathematical models can be used to relate the evolving dynamics of infection and biological mechanisms of transmission at the population level^19,20^. By using optimal control techniques, Getachew et al.^21^ built a deterministic mathematical model of typhoid disease to investigate its effects. The author also utilized some control strategies with cost-effective approaches. Musa et al.^22^ inspected the spread dynamics of the typhoid fever epidemic. The model evaluates how public health education initiatives affect the pathogenesis of typhoid fever, which can lead to significant outbreaks, especially in areas with limited resources. To analyze the dynamics of typhoid fever sickness while considering infection resistance, a mathematical model was formed by Nthiiri et al.^23^ using a set of ordinary differential equations. Adeboye et al.^24^ constructed and investigated a mathematical model of typhoid and malaria co-infection that tackles the control of the spread of malaria and typhoid simultaneously. Pitzer et al.^25^ considered a parsimonious age-structured mathematical model of typhoid fever to estimate the initial and indirect impact of vaccination. Cook et al.^26^ examined a mathematical model that discusses both direct and indirect vaccine safety as well as the benefits of a widespread vaccination programme.

Recently, Karunditu et al.^27^ investigated a nonlinear epidemic model for typhoid disease transmission. The stability of the disease-free and endemic equilibria was thoroughly examined for the continuous model. The main intention of this work is to use two finite difference schemes, i.e. RK-4 and NSFD schemes to evaluate various properties of the continuous model to exhibit its sustainability and biological viability. The RK-4 scheme cannot exactly maintain the basic dynamical aspects of the continuous model, resulting in numerical solutions that change from the solutions of the original system. On the other hand, our findings demonstrate that the NSFD method is not only suitable for the continuous model but also yields incredibly accurate and efficient outcomes. Mickens^28^ actually came up with the concept for this. He proposed the name NSFD scheme to differentiate the new numerical system from the previous SFD scheme. In order to compensate for the shortcomings of the RK-4 scheme, the NSFD scheme was developed.

The present paper is structured as follows: The mathematical model is given in Sect.  2. In Sect.  3, the equilibria and basic reproduction number are provided for the model. In Sect.  4, we utilized the RK-4 scheme for the nonlinear mathematical model. We consider the advanced NSFD scheme in Sect.  5 to solve a variety of problems. Using the Schur-Cohn criterion, the local stability of disease-free equilibrium point is examined. However, the Routh-Hurwitz condition is engaged to validate the local stability of the endemic equilibrium point for the NSFD scheme. The global stability of endemic and disease-free equilibria is examined employing the properties of the Lyapunov function. In order to explain our analytical conclusions, numerical simulations are provided in each section. To summarize the whole paper, the conclusions are given in the final section.

## Mathematical model and parameters explanation

**Figure1:**
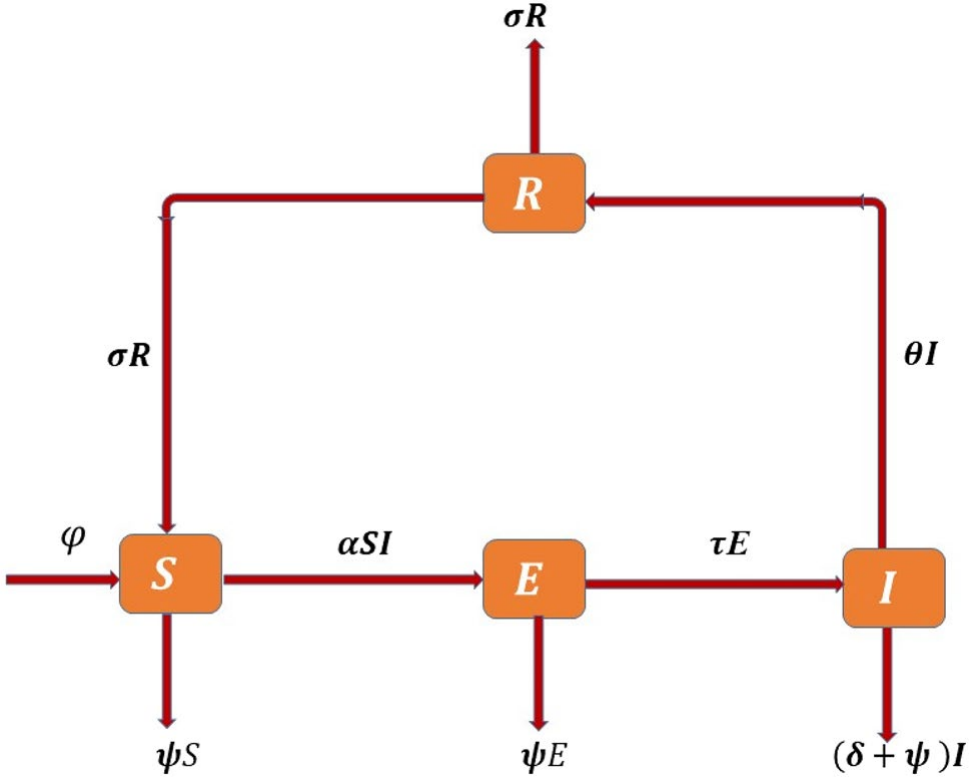
The detailed description of epidemic model for typhoid fever disease transmission.

The present paper discusses and evaluates a deterministic model for the dynamics of typhoid disease. We presume that the whole population $N(t)$ is separated into four sections: Susceptible ($S$), Exposed ($E$), Infected ($I$), and Recovered ($R$), i.e. $N(t)$=$S(t)+E(t)+I(t)+R(t)$. The model employs the subsequent procedure as: $S\to E\to I\to R$. A fractional map for SEIR model of typhoid disease transmission among unprotected people compartments is shown in Fig. 1.

From Fig. 1, we can describe the following SEIR disease system^27^ of nonlinear ordinary differential equations as:

1 $$\begin{aligned} \frac{dS}{{dt}} & = \varphi + \sigma R - \alpha SI - \psi S, \\ \frac{dE}{{dt}} & = \alpha SI - \tau E - \psi E, \\ \frac{dI}{{dt}} & = \tau E - \theta I - \delta I - \psi I, \\ \frac{dR}{{dt}} & = \theta I - \sigma R - \psi R. \\ \end{aligned}$$

### Parameters

The following are descriptions of the parameters used in model (1).

1. $$\psi \,{\text{The rate of innate dying}}$$
2. $$\delta \,{\text{The number of people who die as a result of an illness}}{.}$$
3. $$\varphi \,{\text{The pace of human recruiting }}\left( {{\text{birth}}} \right)$$
4. $$\alpha \,{\text{The rate of disease interaction}}$$
5. $$\tau \,{\text{The rate of unprotected symptoms}}\,$$
6. $$\theta \,{\text{The rate of infectious recovery}}$$
7. $$\sigma \,{\text{The rate at which recovered humans loses temporary immunity}}$$

The parameters $\psi ,\delta ,\varphi ,\alpha ,\tau ,\theta ,\sigma$ are all positive constants. As we know that.

$$N = S + E + I + R.$$

Consequently, by combining all dynamical equations in system (1), we get.

2 $$\frac{dN}{{dt}} = \varphi - \psi S - \psi E - \delta I - \psi R - \psi I.$$

From Eq. (2), we can write

$$\frac{dN}{dt}\le \varphi -\psi S$$

and.

$$\mathop {\lim }\limits_{t \to \infty } {\text{Sup}}N \le \frac{\varphi }{\psi }.$$

Therefore, the feasible area for the continuous system (1) becomes.

3 $$A = \left\{ {\left( {S,E,I,R} \right) \in R^{4} ,S + E + I + R \le \frac{\varphi }{\psi }} \right\}.$$

## Equilibrium points and basic reproductive number

In this section, we establish the equilibrium points of the model (1) and basic reproductive number.

### Equilibrium points

For system (1), there exist the following two nonnegative equilibria.

1. Disease-free equilibrium (DFE) point ${E}^{0}=\left({S}^{0},{E}^{0},{I}^{0},{R}^{0}\right)=\left(\frac{\varphi }{\psi },\mathrm{0,0},0\right).$
2. Disease endemic equilibrium (DEE) point $${E}^{*}$$= ($${S}^{*},{E}^{*},{I}^{*}{,R}^{*})$$,

where

$$\begin{aligned} S^{*} & = \frac{{\left( {\tau + \psi } \right)\left( {\theta + \delta + \psi } \right)}}{\alpha \tau },\;\;E^{*} = \frac{{\left( {\theta + \delta + \psi } \right)\left( {\sigma + \psi } \right)\left( {\varphi \tau \alpha - \psi \left( {\tau + \psi } \right)\left( {\theta + \delta + \psi } \right)} \right)}}{{\alpha \tau \left\{ {\left( {\sigma + \psi } \right)\left( {\tau + \psi } \right)\left( {\theta + \delta + \psi } \right)} \right\} - \theta \tau \sigma }} \\ I^{*} & = \frac{{\left( {\sigma + \psi } \right)}}{\alpha }\left( {\frac{{\varphi \tau \alpha - \psi \left\{ {\left( {\tau + \psi } \right)\left( {\theta + \delta + \psi } \right)} \right\}}}{{\left( {\sigma + \psi } \right)\left( {\tau + \psi } \right)\left( {\theta + \delta + \psi } \right) - \theta \tau \sigma }}} \right),\;\;R^{*} = \frac{1}{\alpha }\frac{{\left( {\theta \varphi \tau \alpha - \theta \psi \left( {\left( {\tau + \psi } \right)\left( {\theta + \delta + \psi } \right)} \right)} \right)}}{{\left( {\left( {\sigma + \psi } \right)\left( {\tau + \psi } \right)\left( {\theta + \delta + \psi } \right) - \theta \tau \sigma } \right)}} \\ \end{aligned}$$

### Basic reproductive number ${({\varvec{R}}}_{0})$

The fundamental reproductive number ${(R}_{0}$), which measures the average rate of latest cases in a residents that is perfectly susceptible, is a critical threshold value in epidemiology. To find the reproductive number, we utilize the idea of next generation matrix provided by the authors in^29^. For typhoid fever disease model (1), we can easily get

$$R_{0} = \frac{{\alpha \varphi \tau }}{{\psi (\tau + \psi )(\theta + \delta + \psi )}}.$$

In the following two sections, a comparison between NSFD scheme and RK-4 is provided. As our main concern is NSFD scheme, therefore the advantages and uses of NSFD are discussed in detail. Specifically, traditional issues concerned to the behavior of these schemes, i.e. equilibrium points, positivity and stability are discussed with respect to increment of the time step.

## The RK-4 scheme

The RK-4 scheme^30^ is widely used approach to solve a system of ordinary differential equations. In numerous situations, we frequently employ the RK-4 scheme, unless stated otherwise. Let $S={H}_{i},E={L}_{i},I={P}_{i},R={Q}_{i}$ for $i=\mathrm{1,2},\mathrm{3,4}$, then system (1) may be signified using the RK-4 scheme as given below.

Stage -1

$$\begin{aligned} H_{1} & = h \left[ {\varphi + \sigma R_{n} - \alpha S_{n} I_{n} - \psi S_{n} } \right] \\ L_{1} & = h \left[ {\alpha S_{n} I_{n} - \tau E_{n} - \psi E_{n} } \right] \\ P_{1 } & = h \left[ {\tau E_{n} - \theta I_{n} - \delta I_{n} - \psi I_{n} } \right] \\ Q_{1} & = h \left[ {\theta I_{n} - \sigma R_{n} - \psi R_{n} } \right] \\ \end{aligned}$$

Stage-2

$$\begin{aligned} H_{2} & = h\left[ {\varphi + \sigma \left( {R_{n} + \frac{{Q_{1} }}{2}} \right) - \alpha \left( {S_{n} + \frac{{H_{1} }}{2}} \right)\left( {I_{n} + \frac{{P_{1} }}{2}} \right) - \psi \left( {S_{n} + \frac{{H_{1} }}{2}} \right)} \right] \\ L_{2} & = h\left[ {\alpha \left( {S_{n} + \frac{{H_{1} }}{2}} \right)\left( {I_{n} + \frac{{P_{1} }}{2}} \right) - \tau \left( {E_{n} + \frac{{L_{1} }}{2}} \right) - \psi \left( {E_{n} + \frac{{L_{1} }}{2}} \right)} \right] \\ P_{2} & = h\left[ {\tau \left( {E_{n} + \frac{{L_{1} }}{2}} \right) - \theta \left( {I_{n} + \frac{{P_{1} }}{2}} \right) - \delta \left( {I_{n} + \frac{{P_{1} }}{2}} \right) - \psi \left( {I_{n} + \frac{{P_{1} }}{2}} \right)} \right] \\ Q_{2} & = h\left[ {\theta \left( {I_{n} + \frac{{P_{1} }}{2}} \right) - \sigma \left( {R_{n} + \frac{{Q_{1} }}{2}} \right) - \psi \left( {R_{n} + \frac{{Q_{1} }}{2}} \right)} \right]. \\ \end{aligned}$$

Stage-3

$$\begin{gathered} H_{3} = h[\varphi + \sigma \left( {R_{n} + \frac{{Q_{2} }}{2}} \right) - \alpha \left( {S_{n} + \frac{{H_{2} }}{2}} \right)\left( {I_{n} + \frac{{P_{2} }}{2}} \right) - \psi \left( {S_{n} + \frac{{H_{2} }}{2}} \right) \hfill \\ L_{3} = h[\alpha \left( {S_{n} + \frac{{H_{2} }}{2}} \right)\left( {I_{n} + \frac{{P_{2} }}{2}} \right) - \tau \left( {E_{n} + \frac{{L_{2} }}{2}} \right) - \psi \left( {E_{n} + \frac{{L_{2} }}{2}} \right) \hfill \\ P_{3} = h\left[ {\tau \left( {E_{n} + \frac{{L_{2} }}{2}} \right) - \theta \left( {I_{n} + \frac{{P_{2} }}{2}} \right) - \delta \left( {I_{n} + \frac{{P_{2} }}{2}} \right) - \psi \left( {I_{n} + \frac{{P_{2} }}{2}} \right)} \right] \hfill \\ Q_{3} = h\left[ {\theta \left( {I_{n} + \frac{{P_{2} }}{2}} \right) - \sigma \left( {R_{n} + \frac{{Q_{2} }}{2}} \right) - \psi \left( {R_{n} + \frac{{Q_{2} }}{2}} \right)} \right]. \hfill \\ \end{gathered}$$

Stage-4

$$\begin{aligned} H_{4} & = h\left[ {\varphi + \sigma \left( {R_{n} + Q_{3} } \right) - \alpha \left( {S_{n} + H_{3} } \right)\left( {I_{n} + P_{3} } \right) - \psi \left( {S_{n} + H_{3} } \right)} \right] \\ N_{4} & = h\left[ {\alpha \left( {S_{n} + H_{3} } \right)\left( {I_{n} + P_{3} } \right) - \tau \left( {E_{n} + L_{3} } \right) - \psi \left( {E_{n} + L_{3} } \right)} \right] \\ P_{4} & = h\left[ {\tau \left( {E_{n} + L_{3} } \right) - \theta \left( {I_{n} + P_{3} } \right) - \delta \left( {I_{n} + P_{3} } \right) - \psi \left( {I_{n} + P_{3} } \right)} \right] \\ Q_{4} & = h\left[ {\theta \left( {I_{n} + P_{3} } \right) - \sigma \left( {R_{n} + Q_{3} } \right) - \psi \left( {R_{n} + Q_{3} } \right)} \right] \\ \end{aligned}$$

Thus general form is

$$\begin{aligned} y_{n + 1 } & = y_{n} + \Delta y \\ S_{n + 1} & = S_{n} + \frac{1}{6}\left\{ {H_{1} + 2H_{2} + 2H_{3} + H_{4} } \right\} \\ E_{n + 1} & = E_{n} + \frac{1}{6}\left\{ {L_{1} + 2L_{2} + 2L_{3} + L_{4} } \right\} \\ I_{n + 1 } & = I_{n} + \frac{1}{6}\left\{ {P_{1} + 2P_{2} + 2P_{3} + P_{4} } \right\} \\ R_{n + 1} & = R_{n} + \frac{1}{6}\left\{ {Q_{1} + 2Q_{2} + 2Q_{3} + Q_{4} } \right\} \\ \end{aligned}$$

**Figure 2:**
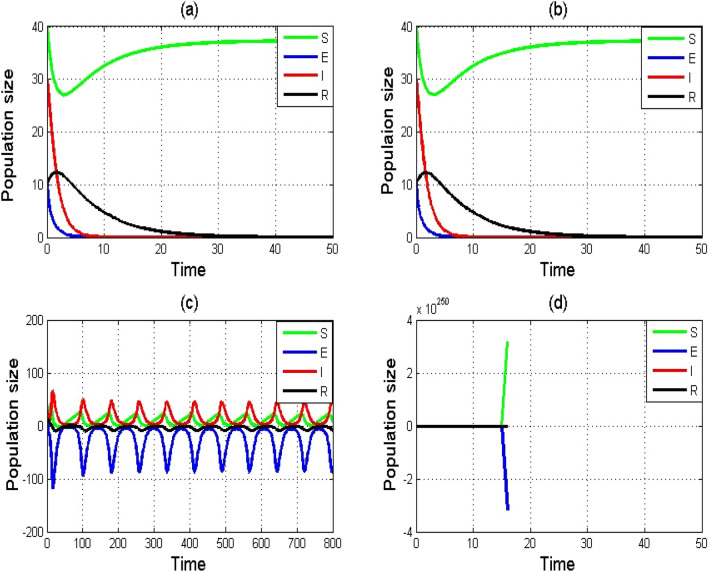
Numerical simulation of SEIR model $\left(1\right)$ by using RK-4 scheme with (**a**) $h$=0.01, (**b**) $h$=0.1, (**c**) $h$=0.9, and (**d**) $h$=1. Remaining parameters are set as $\varphi =0.75,\tau =1.99,\psi =0.02,\theta =0.1503,\alpha =0.0125, \delta =0.625,\sigma =0.125.$

The RK-4 approach is graphically depicted in Fig. 2a–d with various step sizes. The RK-4 approach clearly yields stable and positive solutions for small step sizes, as seen in Fig. 2a,b. As the step size is increased, the equilibrium point stability is shattered for model (1), as illustrated in Fig. 2c,d. Hence, we conclude that the RK-4 technique cannot be employed for large step sizes.

## The NSFD scheme

In this section, our main objective is to discuss the dynamics of NSFD scheme for model (1). The NSFD scheme concept was presented by Mickens in 1994^28^. The NSFD scheme is an iterative method in which we move closer to a solution through iterations^31^. The NSFD scheme is a valuable technique used to solve problems in epidemiology^32–36^, ecology^37,38^, and meta-population modeling^39^. The following will show that, despite the step size $h$, the discrete NSFD scheme sustains the dynamical properties of the corresponding continuous model (1).

### Construction of NSFD scheme

For model (1), we use the notation ${S}_{n}$, ${E}_{n},{ I}_{n}$ and ${R}_{n}$ to indicate the numerical estimates of $S\left(t\right),E(t),I(t)$ and $R\left(t\right)$ at time step $t=nh$, where $h$ is nonnegative time step size and $n$ is a nonnegative integer^40^.

4 $$\begin{aligned} \frac{{S_{n + 1} - S_{n} }}{h} & = \varphi + \sigma R_{n} - \alpha S_{n + 1} I_{n} - \psi S_{n + 1} \\ \frac{{E_{n + 1} - E_{n} }}{h} & = \alpha S_{n + 1} I_{n} - \tau E_{n + 1} - \psi E_{n + 1} \\ \frac{{I_{n + 1} - I_{n} }}{h} & = \tau E_{n + 1} - \theta I_{n + 1} - \delta I_{n + 1} - \psi I_{n + 1} \\ \frac{{R_{n + 1} - R_{n} }}{h} & = \theta I_{n + 1} - \sigma R_{n + 1} - \psi R_{n + 1} \\ \end{aligned}$$

It is assumed that the starting quantities of NSFD SEIR model (4) are also nonnegative. From (4), we get

5 $$\begin{aligned} S_{n + 1} & = \frac{{S_{n} + h\varphi + h\alpha R_{n} }}{{1 + h\left( {\alpha I_{n} + \psi } \right)}} \\ E_{n + 1} & = \frac{{E_{n} + h\alpha S_{n + 1} I_{n} }}{ 1 + h\psi + h\tau } \\ I_{n + 1} & = \frac{{I_{n} + h\tau E_{n + 1} }}{1 + h\theta + h\delta + h\psi } \\ R_{n + 1} & = \frac{{R_{n} + h\theta I_{n + 1} }}{1 + h\sigma + h\psi }. \\ \end{aligned}$$

In the same way like continuous model (1), we can determine a feasible region for the discrete scheme (5). If we indicate ${N}_{n}={S}_{n}+{E}_{n}+{I}_{n}+{R}_{n}$, then by combining all the four equations in system (4) we obtain

$$\frac{{N}_{n+1}-{N}_{n}}{h}=\varphi -\psi {N}_{n+1}\iff \left(1+h\psi \right){N}_{n+1}=h\varphi +{N}_{n}$$

and.

$$N_{n + 1} \le \frac{h\varphi }{{1 + h\psi }} + \frac{{N_{n} }}{1 + h\psi } \Leftrightarrow h\psi \mathop \sum \limits_{j + 1}^{n} \left( {\frac{1}{1 + h\psi }} \right)^{j} + N_{0} \left( {\frac{1}{1 + h\psi }} \right)^{n} .$$

By employing the discrete Gronwall inequality^41–43^, if $0<N(0)<\frac{\varphi }{\psi }$ then.

$$N_{n} \le \frac{\varphi }{\psi }\left( {1 - \frac{1}{{\left( {1 + h\psi } \right)^{n} }}} \right) + N_{0} \left( {\frac{1}{1 + h\psi }} \right)^{n} = \frac{\varphi }{\psi } + \left( {N_{0} - \frac{\varphi }{\psi }} \right)\left( {\frac{1}{1 + h\psi }} \right)^{n} .$$

Since $\left(\frac{1}{1+h\psi }\right)<1$, so we get ${N}_{n}\to \frac{\varphi }{\psi }$ as $n\to \infty$. Therefore, the feasible region for NSFD scheme (5) becomes.

6 $$\overline{A} = \left\{ {\left( {S_{n} ,E_{n} ,I_{n} ,R_{n} } \right):0 \le S_{n} + E_{n} + I_{n} + R_{n} \le \frac{\varphi }{\psi }} \right\}.$$

In the following, we provide the stability conditions of DFE and DEE points for the discrete NSFD scheme (5). We first describe the local stability of both equilibrium points in order to achieve this goal.

### Local stability for NSFD Scheme

To prove that DFE and DEE points are locally asymptotically stable (LAS), we consider

7 $$\begin{aligned} S_{n + 1} & = \frac{{S_{n} + h\varphi + h\alpha R_{n} }}{{1 + h\left( {\alpha I_{n} + \psi } \right)}} = F_{1} \\ E_{n + 1} & = \frac{{E_{n} + h\alpha S_{n + 1} I_{n} }}{{ 1 + h\left( {\psi + \tau } \right)}} = F_{2} \\ I_{n + 1} & = \frac{{I_{n} + h\tau E_{n + 1} }}{{1 + h\left( {\theta + \delta + \psi } \right)}} = F_{3} \\ R_{n + 1} & = \frac{{R_{n} + h\theta I_{n + 1} }}{{1 + h\left( {\sigma + \psi } \right)}} = F_{4} \\ \end{aligned}$$

To demonstrate that DFE and DEE points are LAS, as stated in Lemma 1, we shall utilize the Schur-Cohn condition^44,45^.

#### Lemma 1

The roots of equation ${\lambda }^{2}-T\lambda +D=0$ guarantee $|{\lambda }_{i}|<1$, $i$=1,2 if and only if the requirements listed below are met.

i. $$D < 1$$
ii. $$1 + D + T > 0$$
iii. $$1 - T + D > 0$$

where $T$ and $D$ stand for the Jacobian matrix trace and determinant, respectively.

#### Theorem 1

For all $h>0$, the DFE point ${E}^{0}$ of the NSFD model (5) is LAS whenever ${R}_{0}<1$.

#### Proof

Let us consider the Jacobian matrix.

8 $$J = \left[ {\begin{array}{*{20}c} {\frac{{\partial F_{1} }}{\partial S}} & {\frac{{\partial F_{1} }}{\partial E}} & {\frac{{\partial F_{1} }}{\partial I}} & {\frac{{\partial F_{1} }}{\partial R}} \\ {\frac{{\partial F_{2} }}{\partial S}} & {\frac{{\partial F_{2} }}{\partial E}} & {\frac{{\partial F_{2} }}{\partial I}} & {\frac{{\partial F_{2} }}{\partial R}} \\ {\frac{{\partial F_{3} }}{\partial S}} & {\frac{{\partial F_{3} }}{\partial E}} & {\frac{{\partial F_{3} }}{\partial I}} & {\frac{{\partial F_{3} }}{\partial R}} \\ {\frac{{\partial F_{4} }}{\partial S}} & {\frac{{\partial F_{4} }}{\partial E}} & {\frac{{\partial F_{4} }}{\partial E}} & {\frac{{\partial F_{4} }}{\partial R}} \\ \end{array} } \right].$$

In the following, we first find all the derivatives used in (8).

$$\begin{aligned} \frac{{\partial F_{1} }}{\partial S} & = \frac{1}{{1 + h\left( {\alpha I_{n} + \psi } \right)}},\frac{{\partial F_{1} }}{\partial E} = 0,\frac{{\partial F_{1} }}{\partial I} = \frac{{ - h\alpha \left( { S_{n} + h\varphi + h\alpha R_{n + 1} } \right)}}{{\left( {1 + h\left( {\alpha I_{n} + \psi } \right)} \right)^{2} }},\frac{{\partial F_{1} }}{\partial R} = \frac{h\alpha }{{1 + h\left( {\alpha I_{n} + \psi } \right)}},\\ \frac{{\partial F_{2} }}{\partial S} &= \frac{{h\alpha I_{n} }}{1 + h\psi + h\tau }, \frac{{\partial F_{2} }}{\partial E} = \frac{1}{{1 + h\left( {\psi + \tau } \right)}},\frac{{\partial F_{2} }}{\partial I} = \frac{{h\alpha S_{n + 1} }}{{1 + h\left( {\psi + \tau } \right)}},\frac{{\partial F_{2} }}{\partial R} = 0,\frac{{\partial F_{3} }}{\partial S} = 0,\\ \frac{{\partial F_{3} }}{\partial E} & = \frac{h\tau }{{1 + h\left( {\psi + \theta + \delta } \right)}}, \frac{{\partial F_{3} }}{\partial I} = \frac{1}{{1 + h\left( {\psi + \theta + \delta } \right)}}, \frac{{\partial F_{3} }}{\partial R} = 0,\frac{{\partial F_{4} }}{\partial S} = 0,\frac{{\partial F_{4} }}{\partial E} = 0, \\ \frac{{\partial F_{4} }}{\partial I} & = \frac{h\theta }{{1 + h\left( {\sigma + \psi } \right)}}, \frac{{\partial F_{4} }}{\partial R} = \frac{1}{{1 + h\left( {\sigma + \psi } \right)}}. \\ \end{aligned}$$

By substituting the values of all the derivatives in Eq. (8), we get.

$$J = \left[ {\begin{array}{*{20}c} {\frac{1}{{1 + h\left( {\alpha I_{n} + \psi } \right)}}} & 0 & {\frac{{ - h\alpha \left( { S_{n} + h\varphi + h\alpha R_{n + 1} } \right)}}{{\left( {1 + h\left( {\alpha I_{n} + \psi } \right)} \right)^{2} }}} & {\frac{h\alpha }{{1 + h\left( {\alpha I_{n} + \psi } \right)}}} \\ {\frac{{h\alpha I_{n} }}{1 + h\psi + h\tau }} & {\frac{1}{{1 + h\left( {\psi + \tau } \right)}}} & {\frac{{h\alpha S_{n + 1} }}{{1 + h\left( {\psi + \tau } \right)}}} & 0 \\ 0 & {\frac{h\tau }{{1 + h\left( {\psi + \theta + \delta } \right)}}} & {\frac{1}{{1 + h\left( {\psi + \theta + \delta } \right)}}} & 0 \\ 0 & 0 & {\frac{h\theta }{{1 + h\left( {\sigma + \psi } \right)}}} & {\frac{1}{{1 + h\left( {\sigma + \psi } \right)}}} \\ \end{array} } \right].$$

The aforementioned matrix becomes at DFE point ${E}^{0}=(\frac{\varphi }{\psi },\mathrm{0,0},0)$

$$J\left( {E^{0} } \right) = \left[ {\begin{array}{*{20}c} {\frac{1}{1 + h\psi }} & 0 & {\frac{{ - h\alpha \left( { \frac{\varphi }{\psi } + h\varphi } \right)}}{{(1 + h\psi )^{2} }}} & {\frac{h\alpha }{{1 + h\psi }}} \\ 0 & {\frac{1}{{1 + h\left( {\psi + \tau } \right)}}} & {\frac{{h\alpha \frac{\varphi }{\psi }}}{{1 + h\left( {\psi + \tau } \right)}}} & 0 \\ 0 & {\frac{h\tau }{{1 + h\left( {\psi + \theta + \delta } \right)}}} & {\frac{1}{{1 + h\left( {\psi + \theta + \delta } \right)}}} & 0 \\ 0 & 0 & {\frac{h\theta }{{1 + h\left( {\sigma + \psi } \right)}}} & {\frac{1}{{1 + h\left( {\sigma + \psi } \right)}}} \\ \end{array} } \right].$$

To determine the eigenvalues, we take into consideration.

$$\left| {\begin{array}{*{20}c} {\frac{1}{1 + h\psi } - \lambda } & 0 & {\frac{{ - h\alpha \left( {\frac{\varphi }{\psi } + h\varphi } \right)}}{{(1 + h\psi )^{2} }}} & {\frac{h\alpha }{{1 + h\psi }}} \\ 0 & {\frac{1}{{1 + h\left( {\psi + \tau } \right)}} - \lambda } & {\frac{{h\alpha \frac{\varphi }{\psi }}}{{1 + h\left( {\psi + \tau } \right)}}} & 0 \\ 0 & {\frac{h\tau }{{1 + h\left( {\psi + \theta + \delta } \right)}}} & {\frac{1}{{1 + h\left( {\psi + \theta + \delta } \right)}} - \lambda } & 0 \\ 0 & 0 & {\frac{h\theta }{{1 + h\left( {\sigma + \psi } \right)}}} & {\frac{1}{{1 + h\left( {\sigma + \psi } \right)}} - \lambda } \\ \end{array} } \right| = 0.$$

The above equation can be rewritten as.

9 $$\left( {\frac{1}{1 + h\psi } - \lambda } \right)\left( {\frac{1}{{1 + h\left( {\sigma + \psi } \right)}} - \lambda } \right)\left| {\begin{array}{*{20}c} {\frac{1}{{1 + h\left( {\psi + \tau } \right)}} - \lambda } & {\frac{{h\alpha \frac{\varphi }{\psi }}}{{1 + h\left( {\psi + \tau } \right)}}} \\ {\frac{h\tau }{{1 + h\left( {\psi + \theta + \delta } \right)}}} & {\frac{1}{{1 + h\left( {\psi + \theta + \delta } \right)}} - \lambda } \\ \end{array} } \right| = 0.$$

The Eq. (9) gives ${\lambda }_{1}=\frac{1}{1+h\psi }<1,{\lambda }_{2}=\frac{1}{1+h\left(\sigma +\psi \right)}<1$. To discuss the other two eigenvalues, we consider.

10 $$\left| {\begin{array}{*{20}c} {\frac{1}{{1 + h\left( {\psi + \tau } \right)}} - \lambda } & {\frac{{h\alpha \frac{\varphi }{\psi }}}{{1 + h\left( {\psi + \tau } \right)}}} \\ {\frac{h\tau }{{1 + h\left( {\psi + \theta + \delta } \right)}}} & {\frac{1}{{1 + h\left( {\psi + \theta + \delta } \right)}} - \lambda } \\ \end{array} } \right| = 0.$$

From Eq. (10), we obtain.

11 $$\lambda^{2} - \lambda \left( {\frac{1}{{1 + h\left( {\psi + \tau } \right)}} + \frac{1}{{1 + h\left( {\psi + \theta + \delta } \right)}}} \right) + \frac{1}{{\left( {1 + h\left( {\psi + \theta + \delta } \right)} \right)\left( {1 + h\left( {\psi + \tau } \right)} \right)}} - \frac{h\alpha \varphi }{{\psi \left( {1 + h\left( {\psi + \tau } \right)} \right)\left( {1 + h\left( {\psi + \theta + \delta } \right)} \right)}} = 0.$$

Comparing (11) with ${\lambda }^{2}-T\lambda +D=0$, we get $T=\frac{1}{1+h\left(\psi +\tau \right)}+\frac{1}{1+h\left(\theta +\delta +\psi \right)}$ and $D=\frac{1}{\left(1+h\left(\psi +\theta +\delta \right)\right)(1+h\left(\psi +\tau \right))}-\frac{h\alpha \varphi }{\psi (1+h\left(\psi +\tau \right))(1+h\left(\psi +\theta +\delta \right))}$. If ${R}_{0}<1$, i.e. $\alpha \varphi \tau <\psi (\tau +\psi )(\psi +\theta +\delta )$ then all the three conditions of Lemma 1 are satisfied, i.e.

$$\begin{aligned} & D = \frac{1}{{\left( {1 + h\left( {\psi + \theta + \delta } \right)} \right)\left( {1 + h\left( {\psi + \tau } \right)} \right)}} + \frac{h\alpha \varphi }{{\psi \left( {1 + h\left( {\psi + \tau } \right)} \right)\left( {1 + h\left( {\psi + \theta + \delta } \right)} \right)}} < 1. \\ & \quad 1 + T + D = 1 + \frac{1}{{1 + h\left( {\psi + \tau } \right)}} + \frac{1}{{1 + h\left( {\theta + \delta + \psi } \right)}} \\ & \quad + \frac{1}{{\left( {1 + h\left( {\psi + \theta + \delta } \right)} \right)\left( {1 + h\left( {\psi + \tau } \right)} \right)}} - \frac{h\alpha \varphi }{{\psi \left( {1 + h\left( {\psi + \tau } \right)} \right)\left( {1 + h\left( {\psi + \theta + \delta } \right)} \right)}} > 0 \\ & \quad 1 - T + D = 1 - { }\frac{1}{{1 + h\left( {\psi + \tau } \right)}} - \frac{1}{{1 + h\left( {\theta + \delta + \psi } \right)}} \\ & \quad + \frac{1}{{\left( {1 + h\left( {\psi + \theta + \delta } \right)} \right)\left( {1 + h\left( {\psi + \tau } \right)} \right)}} - \frac{h\alpha \varphi }{{\psi \left( {1 + h\left( {\psi + \tau } \right)} \right)\left( {1 + h\left( {\psi + \theta + \delta } \right)} \right)}} > 0. \\ \end{aligned}$$

All of the Schur–Cohn requirements described in Lemma 1 are consequently satisfied whenever ${R}_{0}<1$. As a result, the DFE point ${E}^{0}$ of discrete NSFD scheme (5) is LAS, provided that ${R}_{0}<1$.

When a disease is prevalent in a population, it will continue to exist in that community. In the following theorem, we will use Routh-Hurwitz criterion^46,47^ to examine that DEE point ${E}^{*}$ is LAS.

#### Theorem 2

For all $h>0$, the DEE point ${E}^{*}$ of the NSFD model (5) is LAS whenever ${R}_{0}>1$.

#### Proof

The Jacobian matrix is obtained according to Theorem 1 as.

$$J = \left[ {\begin{array}{*{20}c} {\frac{1}{{1 + h\left( {\alpha I_{n} + \psi } \right)}}} & 0 & {\frac{{ - h\alpha \left( { S_{n} + h\varphi + h\alpha R_{n + 1} } \right)}}{{\left( {1 + h\left( {\alpha I_{n} + \psi } \right)} \right)^{2} }}} & {\frac{h\alpha }{{1 + h\left( {\alpha I_{n} + \psi } \right)}}} \\ {\frac{{h\alpha I_{n} }}{{1 + h\left( {\psi + \tau } \right)}}} & {\frac{1}{{1 + h\left( {\psi + \tau } \right)}}} & {\frac{{h\alpha S_{n + 1} }}{{1 + h\left( {\psi + \tau } \right)}}} & 0 \\ 0 & {\frac{h\tau }{{1 + h\left( {\psi + \theta + \delta } \right)}}} & {\frac{1}{{1 + h\left( {\psi + \theta + \delta } \right)}}} & 0 \\ 0 & 0 & {\frac{h\theta }{{1 + h\left( {\sigma + \psi } \right)}}} & {\frac{1}{{1 + h\left( {\sigma + \psi } \right)}}} \\ \end{array} } \right].$$

By putting DEE point ${E}^{*}$, we obtain.

$$J\left( {E^{*} } \right) = \left[ {\begin{array}{*{20}c} {\frac{1}{{1 + h\left( {\alpha I^{*}_{n} + \psi } \right)}}} & 0 & {\frac{{ - h\alpha \left( { S^{*}_{n} + h\varphi + h\alpha R^{*}_{n + 1} } \right)}}{{\left( {1 + h\left( {\alpha I^{*}_{n} + \psi } \right)} \right)^{2} }}} & {\frac{h\alpha }{{1 + h\left( {\alpha I^{*}_{n} + \psi } \right)}}} \\ {\frac{{h\alpha I^{*}_{n} }}{1 + h\psi + h\tau }} & {\frac{1}{{1 + h\left( {\psi + \tau } \right)}}} & {\frac{{h\alpha S^{*}_{n + 1} }}{{1 + h\left( {\psi + \tau } \right)}}} & 0 \\ 0 & {\frac{h\tau }{{1 + h\left( {\psi + \theta + \delta } \right)}}} & {\frac{1}{{1 + h\left( {\theta + \delta + \psi } \right)}}} & 0 \\ 0 & 0 & {\frac{h\theta }{{1 + h\left( {\sigma + \psi } \right)}}} & {\frac{1}{{1 + h\left( {\sigma + \psi } \right)}}} \\ \end{array} } \right].$$

To determine the eigenvalues, we take into consideration.

$$\left| {\begin{array}{*{20}c} {\frac{1}{{1 + h\left( {\alpha I^{*}_{n} + \psi } \right)}} - \lambda } & 0 & {\frac{{ - h\alpha \left( {S^{*}_{n} + h\varphi + h\alpha R^{*}_{n + 1} } \right)}}{{\left( {1 + h\left( {\alpha I^{*}_{n} + \psi } \right)} \right)^{2} }}} & {\frac{h\alpha }{{1 + h\left( {\alpha I^{*}_{n} + \psi } \right)}}} \\ {\frac{{h\alpha I^{*}_{n} }}{1 + h\psi + h\tau }} & {\frac{1}{{1 + h\left( {\psi + \tau } \right)}} - \lambda } & {\frac{{h\alpha S^{*}_{n + 1} }}{{1 + h\left( {\psi + \tau } \right)}}} & 0 \\ 0 & {\frac{h\tau }{{1 + h\left( {\psi + \theta + \delta } \right)}}} & {\frac{1}{{1 + h\left( {\psi + \theta + \delta } \right)}} - \lambda } & 0 \\ 0 & 0 & {\frac{h\theta }{{1 + h\left( {\sigma + \psi } \right)}}} & {\frac{1}{{1 + h\left( {\sigma + \psi } \right)}} - \lambda } \\ \end{array} } \right| = 0.$$

The characteristic equation for above equation becomes as.

12 $$\lambda^{4} + P_{1} \lambda^{3} + P_{2} \lambda^{2} + P_{3} \lambda + P_{4} = 0,$$

where

$$\begin{aligned} P_{1} & = \frac{1}{{1 + h\left( {\alpha I^{*}_{n} + \psi } \right)}} + \frac{1}{{1 + h\left( {\psi + \tau } \right)}} + \frac{1}{{1 + h\left( {\sigma + \psi } \right)}} > 0, \\ P_{2} & = \frac{1}{{\left( {1 + h\left( {\psi + \theta + \delta } \right)} \right)\left( {1 + h\left( {\tau + \psi } \right)} \right)}} - \frac{{h^{2} \tau \alpha S_{n + 1}^{*} }}{{\left( {1 + h\left( {\tau + \psi } \right)} \right)\left( {1 + h\left( {\psi + \theta + \delta } \right)} \right)}} \\ & \quad - \left( {\frac{1}{{1 + h\left( {\alpha I^{*}_{n} + \psi } \right)}} + \frac{1}{{1 + h\left( {\sigma + \psi } \right)}}} \right)\left( {\frac{1}{{\left( {1 + h\left( {\psi + \theta + \delta } \right)} \right)\left( {1 + h\left( {\tau + \psi } \right)} \right)}}} \right)\\&\quad + \frac{1}{{\left( {1 + h\left( {\alpha I^{*}_{n} + \psi } \right)} \right)\left( {1 + h\left( {\sigma + \psi } \right)} \right)}} > 0, \\ P_{3} & = \frac{1}{{\left( {1 + h\left( {\alpha I^{*}_{n} + \psi } \right)} \right)}} + \frac{1}{{\left( {1 + h\left( {\sigma + \psi } \right)} \right)\left( {1 + h\left( {\tau + \psi } \right)} \right)\left( {1 + h\left( {\psi + \theta + \delta } \right)} \right)}} \\ & \quad + \frac{{h^{2} \tau \alpha S_{n + 1}^{*} }}{{\left( {1 + h\left( {\alpha I^{*}_{n} + \psi } \right)} \right)\left( {1 + h\left( {\sigma + \psi } \right)} \right)\left( {1 + h\left( {\tau + \psi } \right)} \right)\left( {1 + h\left( {\psi + \theta + \delta } \right)} \right)}} \\ &\quad+ \frac{{h^{2} \tau \alpha }}{{\left( {1 + h\left( {\alpha I^{*}_{n} + \psi } \right)} \right)(1 + h\left( {\alpha I^{*}_{n} + \psi } \right)^{2} }} \\ & \quad + \frac{1}{{\left( {1 + h\left( {\alpha I^{*}_{n} + \psi } \right)} \right)\left( {1 + h\left( {\sigma + \psi } \right)} \right)\left( {1 + h\left( {\tau + \psi } \right)} \right)(1 + h\left( {\psi + \theta + \delta } \right)}} > 0, \\ P_{4} & = \frac{{h^{2} \tau \alpha \left( {S^{*}_{n} + h\varphi + h\alpha R^{*}_{n} } \right)}}{{\left( {1 + h\left( {\alpha I^{*}_{n} + \psi } \right)} \right)\left( {1 + h\left( {\alpha I^{*}_{n} + \psi } \right)} \right)^{2} \left( {1 + h\left( {\sigma + \psi } \right)} \right)}} \\ &\quad+ \frac{{h^{3} \tau \alpha \theta }}{{\left( {1 + h\left( {\sigma + \psi } \right)} \right)\left( {1 + h\left( {\alpha I^{*}_{n} + \psi } \right)} \right)\left( {1 + h\left( {\alpha I^{*}_{n} + \psi } \right)} \right)}} \\ & \quad + \frac{1}{{\left( {1 + h\left( {\alpha I^{*}_{n} + \psi } \right)} \right)\left( {1 + h\left( {\sigma + \psi } \right)} \right)\left( {1 + h\left( {\tau + \psi } \right)} \right)\left( {1 + h\left( {\psi + \theta + \delta } \right)} \right)}}. \\ \end{aligned}$$

From above informations, it is clear that if ${R}_{0}>1$, then

$$\begin{aligned} M_{1} & = P_{1} > 0, \\ M_{2} & = P_{1} P_{2} - P_{3} > 0, \\ M_{3} & = \left| {\begin{array}{*{20}c} {P_{1} } & {P_{3} } & 0 \\ 1 & {P_{2} } & {P_{4} } \\ 0 & {P_{1} } & {P_{3} } \\ \end{array} } \right| = - P_{3}^{2} + P_{1} P_{2} P_{3} - P_{1}^{2} P_{4} = P_{3} M_{2} - P_{1}^{2} P_{4} > 0, \\ M_{4} & = P_{4} M_{3} > 0. \\ \end{aligned}$$

According to the Routh-Hurwitz criteria, all of the solutions to the Eq. (12) must have negative real parts. As a result, the DEE point ${E}^{*}$ of the discrete NSFD scheme (5) is LAS whenever ${R}_{0}>1$.

### Global stability for NSFD Scheme

In the following, we now show that ${R}_{0}$ is a critical value for the global stability. If ${R}_{0}\le 1$, then the DFE point ${E}^{0}$ is globally asymptotically stable (GAS) and when ${R}_{0}>1$, then DEE point ${E}^{*}$ becomes GAS. To discuss the global stability of equilibria, we employ the same criterion used by Vaz et al.^48^.

#### Theorem 3

For all $h>0$, the DFE point ${E}^{0}$ of the NSFD model (5) is GAS whenever ${R}_{0}\le 1$.

#### Proof

From the feasible region (6) discussed for NSFD scheme (5), it is clear that ${S}_{n}+{E}_{n}+{I}_{n}+{R}_{n}\le \frac{\varphi }{\psi }$. If ${E}_{n}={I}_{n}={R}_{n}=0$, then ${S}_{n}\le \frac{\varphi }{\psi }$. Therefore, if we take $\upeta > 0$, then there exists an integer ${n}_{0}$ such that for any $n \ge n_{0} , S_{n + 1} < \frac{\varphi }{\psi } +\upeta$. Consider the sequence ${\left\{w(n)\right\}}_{n=0}^{\infty }$ such that.

$$w\left( n \right) = E_{n} + \frac{\omega }{{C_{3} }}I_{n} + \frac{\alpha }{{C_{2} }}R_{n} + h\alpha S_{n + 1} I_{n} ,$$

where ${C}_{2}=\left(\sigma +\psi \right)$ and ${C}_{3}=\left(\theta +\delta +\psi \right)$. For $n\ge {n}_{0}$, we have

$$\begin{aligned} w\left( {n + 1} \right) - w\left( n \right) & = E_{n + 1} + \frac{\omega }{{C_{3} }}I_{n + 1} + \frac{\alpha }{{C_{2} }}R_{n + 1} + h\alpha S_{n + 2} I_{n + 1} - E_{n} - \frac{\omega }{{C_{3} }}I_{n} - \frac{\alpha }{{C_{2} }}R_{n} - h\alpha S_{n + 1} I_{n} \\ & = \left( {E_{n + 1} - E_{n} } \right) + \frac{\omega }{{C_{3} }}\left( {I_{n + 1} - I_{n} } \right) + \frac{\alpha }{{C_{2} }}\left( {R_{n + 1} - R_{n} } \right) + h\alpha S_{n + 2} I_{n + 1} - h\alpha S_{n + 1} I_{n} . \\ \end{aligned}$$

By applying the NSFD scheme (5), we obtain

$$= h\left( {\alpha S_{n + 1} I_{n} - \left( {\tau + \psi } \right)E_{n + 1} } \right) + \frac{\omega }{{C_{3} }}h\left( {\tau E_{n + 1} - (\theta + \delta + \psi )I_{n + 1} } \right) + \frac{\alpha }{{C_{2} }}h\left( {\theta I_{n + 1} - \left( {\sigma + \psi } \right)R_{n + 1} } \right) + h\alpha S_{n + 2} I_{n + 1} - h\alpha S_{n + 1} I_{n} .$$

Let ${C}_{1}=(\tau +\psi )$, then the above expression becomes

$$= h\left( {\alpha S_{n + 2} I_{n} + \left( {\omega \tau - C_{1} C_{3} } \right)\frac{{E_{n + 1} }}{{C_{3} }} + (\frac{\alpha \theta }{{C_{2} }} - \frac{\omega }{{C_{3} }}\left( {\theta + \delta + \psi } \right))I_{n + 1} - \left( {\sigma + \psi } \right)R_{n + 1} } \right).$$

If we put ${C}_{1}{C}_{3}- \omega \tau =D$, the above expression becomes

$$=h\left(\left(\alpha {S}_{n+2}{I}_{n}-D\frac{{E}_{n+1}}{{C}_{3}}\right)-\left(\theta +\delta +\psi \right){I}_{n+1}-\left(\sigma +\psi \right){R}_{n+1}\right).$$

We can choose $\beta$ a very small positive number such that

$$\alpha S_{n + 2} I_{n} \le \beta \left( {E_{n + 1} + C_{3} I_{n + 1} + C_{2} R_{n + 1} } \right) = \beta \left( {E_{n + 1} + C_{3} \frac{\tau }{{C_{3} }}E_{n + 1} + C_{2} \frac{{E_{n + 1} }}{{C_{2} C_{3} }}} \right)$$

Therefore, we get

$$\begin{aligned} w\left( {n + 1} \right) - w\left( n \right) & \le h\left( {\beta \left( {E_{n + 1} + \tau E_{n + 1} + \frac{{E_{n + 1} }}{{C_{3} }}} \right) - D\frac{{E_{n + 1} }}{{C_{3} }}} \right) \\ & \le \frac{{hE_{n + 1} }}{{C_{3} }}\left( {\beta + \beta \frac{{\psi \left( {\tau + \psi } \right)\left( {\theta + \delta + \psi } \right)}}{\alpha \varphi }R_{0} + \beta - D} \right) \\ &= \frac{{hE_{n + 1} }}{{C_{3} }}\left( {2\beta + \frac{{\beta \psi \left( {\tau + \psi } \right)\left( {\theta + \delta + \psi } \right)}}{\alpha \varphi }R_{0} - D} \right). \\ \end{aligned}$$

Let $C=\frac{\psi (\tau +\psi )(\theta +\delta +\psi )}{\alpha \varphi }$, then we get

$$\begin{aligned} & = \frac{{hE_{n + 1} }}{{C_{3} }}\left( {2\beta + C\beta R_{0} - D} \right). \\ & = \frac{{hE_{n + 1} }}{{C_{3} }}\left( {\beta \left( {2 + CR_{0} } \right) - D} \right). \\ \end{aligned}$$

Since $\beta$ is a very small number and $\upeta$ is imprecise Therefore, if ${R}_{0}\le 1$ then we reach the conclusion that for any $n\ge 0$, $0\ge w\left(n+1\right)-w\left(n\right)$ and $\mathop {\lim }\limits_{n \to \infty } I_{n} = 0$. The sequence ${\left\{w(n)\right\}}_{n=0}^{\infty }$ is a monotonically decreasing and $\underset{n\to \infty }{\mathrm{lim}}{S}_{n}=\frac{\varphi }{\psi }$. Therefore, the DFE point ${E}^{0}$ is GAS whenever ${R}_{0}\le 1$.

**Figure 3:**
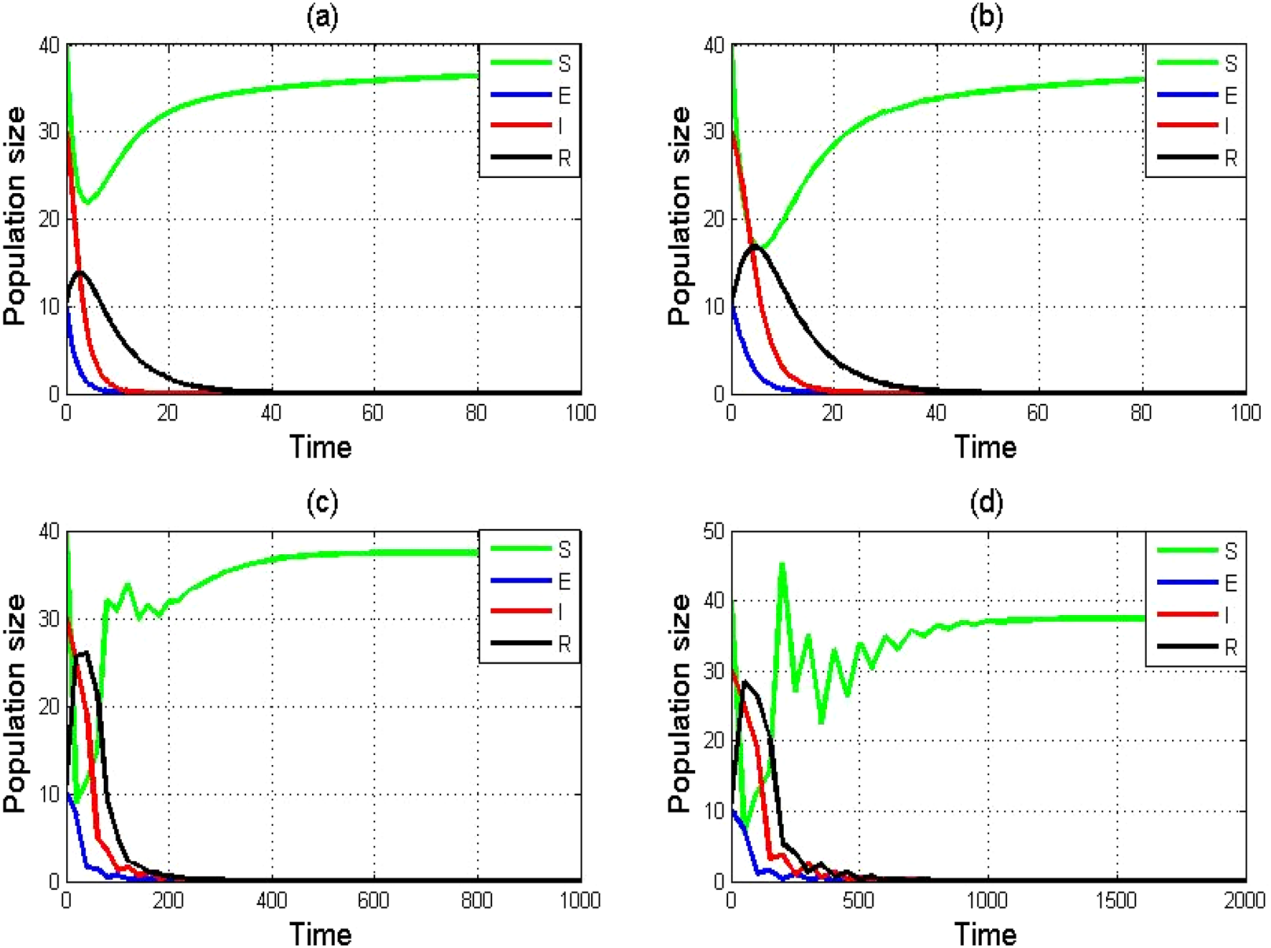
Numerical simulation of SEIR model $\left(1\right)$ by using NSFD scheme with $\left(\mathbf{a}\right)h=0.01,(\mathbf{b}) h=1,(\mathbf{c}) h=20,(\mathbf{d}) h=50$. Remaining parameters are set as $\varphi =0.75,\tau =1.99,\psi =0.02,\theta =0.1503,\alpha =0.0125, \delta =0.625,\sigma =0.125$.

**Figure 4:**
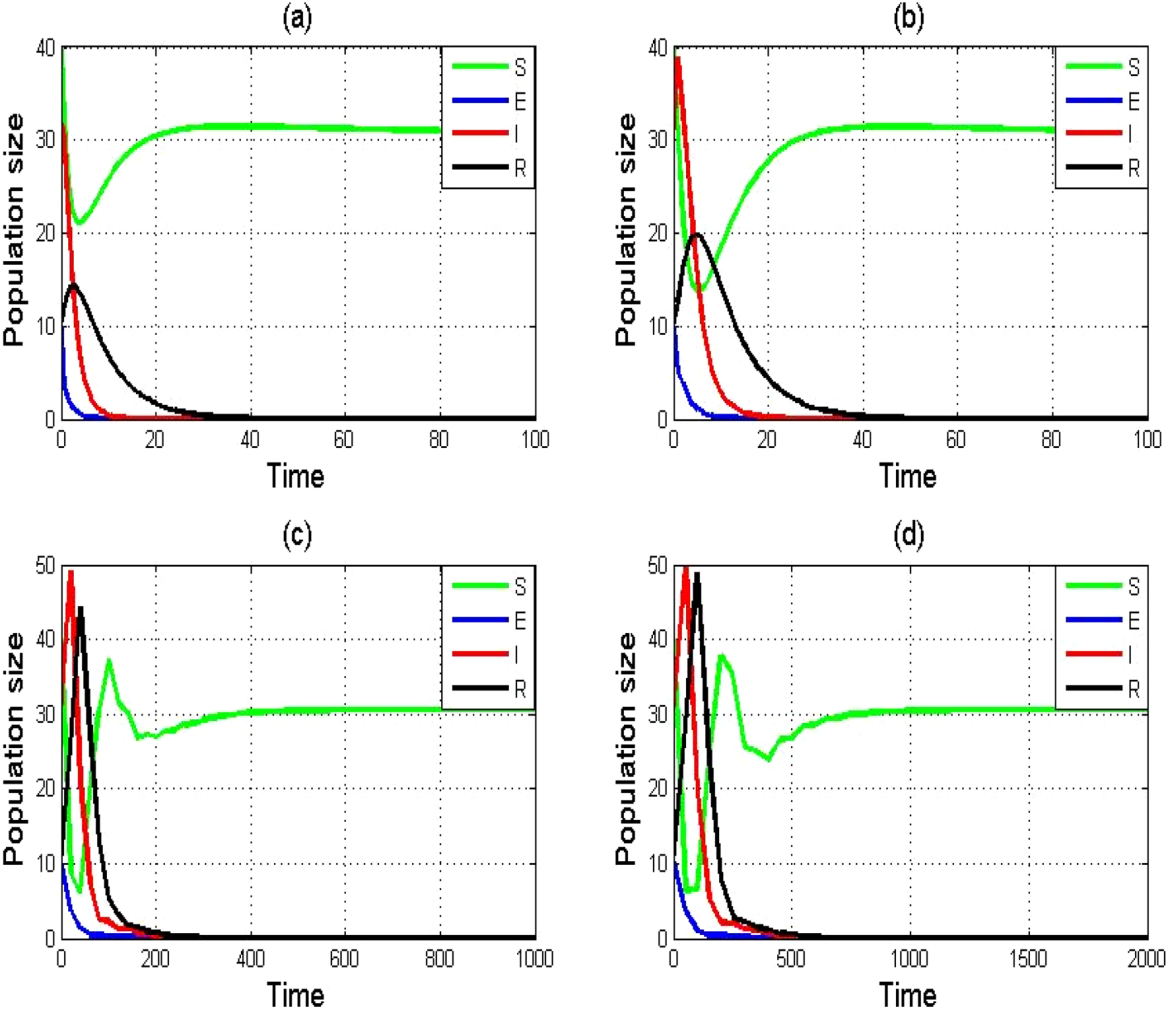
Numerical simulation of SEIR model $\left(1\right)$ by using NSFD scheme with $\left(\mathbf{a}\right)h=0.01,(\mathbf{b}) h=1,(\mathbf{c}) h=20,(\mathbf{d}) h=50$. Remaining parameters are set as $\varphi =0.6112,\tau =4,\psi =0.02,\theta =0.1503,\alpha =0.0125, \delta =0.625,\sigma =0.125$.

The numerical simulations shown in Figs. 3a–d and 4a–d for ${R}_{0}<1$ and ${R}_{0}=1$, respectively also exhibit that the solutions of NSFD scheme (5) converges to the DFE point ${E}^{0}$ independent of the step size. By combining the above two conditions, we conclude that if ${R}_{0}\le 1$ then the DFE point ${E}^{0}$ is GAS for NSFD scheme (5). The NSFD scheme is hence convergent for model (1) for all finite step sizes.

#### Theorem 4

For all $h>0$, the DEE point ${E}^{*}$ of the NSFD model (5) is GAS whenever ${R}_{0}>1$.

#### Proof

Let us construct a sequence ${\left\{w(n)\right\}}_{n=0}^{\infty }$ such that.

$$w\left( n \right) = \frac{1}{{hE^{*} }}p\left( {\frac{{S_{n} }}{{S^{*} }}} \right) + \frac{1}{{hS^{*} }}p\left( {\frac{{E_{n} }}{{E^{*} }}} \right) + \frac{{\omega I^{*} }}{{hC_{3} S^{*} E^{*} }}p\left( {\frac{{I_{n} }}{{I^{*} }}} \right) + \frac{{\alpha R^{*} }}{{hC_{2} S^{*} E^{*} }}p\left( {\frac{{R_{n} }}{{R^{*} }}} \right),$$

Where $p\left(x\right)=x-1-\mathrm{ln}\left(x\right)$ such that $x\in {R}^{+},{ C}_{2}=\sigma +\psi \mathrm{and }{C}_{3}=\theta +\delta +\psi$. It is evident that $p(x)\ge 0$ and if $x=1$, the equality holds. From above, we can write

$$\begin{aligned} p\left( {\frac{{S_{n + 1} }}{{S^{*} }}} \right) - p\left( {\frac{{S_{n} }}{{S^{*} }}} \right) & = \frac{{S_{n + 1} - S_{n} }}{{S^{*} }} - {\text{ln}}\left( {\frac{{S_{n + 1} }}{{S_{n} }}} \right) \\ & \le \frac{{\left( {S_{n + 1} - S^{*} } \right)\left( {S_{n + 1} - S_{n} } \right)}}{{S_{n + 1} S^{*} }} \\ & = \frac{{\left( {S_{n + 1} - S^{*} } \right)}}{{S_{n + 1} S^{*} }}h\left( {\varphi + \sigma R_{n} - \alpha S_{n + 1} I_{n} - \psi S_{n + 1} } \right) \\ & = \frac{{ - \psi h\left( {S_{n + 1} - S^{*} } \right)^{2} }}{{S_{n + 1} S^{*} }} - h\left( {\alpha \left( {1 - \frac{{S^{*} }}{{S_{n + 1} }}} \right)\left( {\frac{{I_{n} }}{{I^{*} }}\frac{{S_{n + 1} }}{{S^{*} }} - 1} \right) - \sigma R_{n} } \right). \\ \end{aligned}$$

In the same way

$$\begin{aligned} p\left( {\frac{{E_{n + 1} }}{{E^{*} }}} \right) - p\left( {\frac{{E_{n} }}{{E^{*} }}} \right) & = \frac{{E_{n + 1} - E_{n} }}{{E^{*} }} - \ln \left( {\frac{{E_{n + 1} }}{{E_{n} }}} \right) \\ & \le \frac{{\left( {E_{n + 1} - E^{*} } \right)\left( {E_{n + 1} - E_{n} } \right)}}{{E_{n + 1} E^{*} }} \\ & = \frac{{\left( {E_{n + 1} - E^{*} } \right)}}{{E_{n + 1} E^{*} }}\left( {\alpha S_{n + 1} I_{n} - \left( {\tau + \psi } \right)E_{n + 1} } \right). \\ \end{aligned}$$

Let $${C}_{1}=\tau +\psi$$, then

$$\begin{aligned} p\left( {\frac{{E_{n + 1} }}{{E^{*} }}} \right) - p\left( {\frac{{E_{n} }}{{E^{*} }}} \right) & \le \left( {\frac{{E_{n + 1} - E^{*} }}{{E^{*} E_{n + 1} }}} \right)\left( {\alpha S_{n + 1} I_{n} - C_{1} E_{n + 1} } \right) \\ & = \left( {\frac{{E_{n + 1} - E^{*} }}{{E^{*} E_{n + 1} }}} \right)\left( {\alpha S_{n + 1} I_{n} - \frac{{\alpha S^{*} E_{n + 1} }}{{E^{*} }}} \right) \\ & = \left( {1 - \frac{{E^{*} }}{{E_{n + 1} }}} \right)\left( {\frac{{h\alpha S^{*} }}{{E^{*} }}} \right)\left( {\frac{{S_{n + 1} }}{{S^{*} }}\frac{{I_{n} }}{{I^{*} }} - \frac{{E_{n + 1} }}{{E^{*} }}} \right), \\ \end{aligned}$$

and

$$\begin{aligned} p\left( {\frac{{I_{n + 1} }}{{I^{*} }}} \right) - p\left( {\frac{{I_{n} }}{{I^{*} }}} \right) & = \left( {\frac{{I_{n + 1} - I_{n} }}{{I^{*} }}} \right) - \ln \left( {\frac{{I_{n + 1} }}{{I_{n} }}} \right) \\ & \quad \le \frac{{\left( {I_{n + 1} - I^{*} } \right)\left( {I_{n + 1} - I_{n} } \right)}}{{I^{*} I_{n + 1} }} \\ & \quad \le \frac{{\left( {I_{n + 1} - I^{*} } \right)}}{{I_{n + 1} I^{*} }}(\tau E_{n + 1} - \left( {\theta + \delta + \psi } \right)I_{n + 1} \\ & \quad \le \frac{{\left( {I_{n + 1} - I^{*} } \right)}}{{I^{*} I_{n + 1} }}\left( {\tau E_{n + 1} - C_{3} I_{n + 1} } \right) \\ = C_{3} h\left( {1 - \frac{{I^{*} }}{{I_{n + 1} }}} \right)\left( {\frac{{E_{n + 1} }}{{E^{*} }} - \frac{{I_{n + 1} }}{{I^{*} }}} \right), \\ \end{aligned}$$

and

$$\begin{aligned} p\left( {\frac{{R_{n + 1} }}{{R^{*} }}} \right) - p\left( {\frac{{R_{n} }}{{R^{*} }}} \right) & = \frac{{R_{n + 1} - R_{n} }}{{R^{*} }} - \ln \left( {\frac{{R_{n + 1} }}{{R_{n} }}} \right) \\ & \quad \le \frac{{\left( {R_{n + 1} - R^{*} } \right)\left( {R_{n + 1} - R_{n} } \right)}}{{R_{n + 1} R^{*} }} \\ & \quad \le \frac{{\left( {R_{n + 1} - R^{*} } \right)}}{{R_{n + 1} R^{*} }}\left( {\theta I_{n + 1} - \left( {\sigma + \psi } \right)R_{n + 1} } \right) \\ & \quad \le \frac{{\left( {R_{n + 1} - R^{*} } \right)}}{{R_{n + 1} R^{*} }}\left( {\theta I_{n + 1} - C_{2} R_{n + 1} } \right) \\ & = C_{2} h\left( {\frac{{I_{n + 1} }}{{I^{*} }} - \frac{{R_{n + 1} }}{{R^{*} }}} \right)\left( {1 - \frac{{R^{*} }}{{R_{n + 1} }}} \right). \\ \end{aligned}$$

The difference of $w(n)$ satisfies

$$\begin{aligned} w\left( {n + 1} \right) - w\left( n \right) & = \frac{1}{{hE^{*} }}\left( {p\left( {\frac{{S_{n + 1} }}{{S^{*} }}} \right) - p\left( {\frac{{S_{n} }}{{S^{*} }}} \right)} \right) + \frac{1}{{hS^{*} }}\left( {p\left( {\frac{{E_{n + 1} }}{{E^{*} }}} \right) - p\left( {\frac{{E_{n} }}{{E^{*} }}} \right)} \right) \\ & \quad + \frac{{\omega I^{*} }}{{hC_{3} S^{*} E^{*} }}\left( {p\left( {\frac{{I_{n + 1} }}{{I^{*} }}} \right) - p\left( {\frac{{I_{n} }}{{I^{*} }}} \right)} \right) + \frac{{\alpha R^{*} }}{{hC_{2} S^{*} E^{*} }}\left( {p\left( {\frac{{R_{n + 1} }}{{R^{*} }}} \right) - p\left( {\frac{{R_{n} }}{{R^{*} }}} \right)} \right) \\ & \quad \le \frac{{ - \alpha h\left( {S_{n + 1} - S^{*} } \right)^{2} }}{{S_{n + 1} S^{*} I^{*} }} - \frac{\alpha }{{E^{*} }}\left( {\frac{{E^{*} }}{{E_{n + 1} }}\frac{{I_{n} }}{{I^{*} }}\frac{{S_{n + 1} }}{{S^{*} }} - 2 - \frac{{I_{n} }}{{I^{*} }} + \frac{{S^{*} }}{{S_{n + 1} }} + \frac{{E_{n + 1} }}{{E^{*} }}} \right) \\ & \quad - \frac{{\omega I^{*} }}{{S^{*} E^{*} }}\left( {\frac{{E^{*} I_{n + 1} }}{{E_{n + 1} I^{*} }} + \frac{{I^{*} E_{n + 1} }}{{I_{n + 1} E^{*} }} - 2} \right) - \frac{{\alpha R^{*} }}{{S^{*} E^{*} }}\left( {\frac{{E^{*} R_{n + 1} }}{{E_{n + 1} R^{*} }} + \frac{{R^{*} E_{n + 1} }}{{R_{n + 1} E^{*} }} - 2} \right) \\ & \quad \le \frac{{ - \alpha h\left( {S_{n + 1} - S^{*} } \right)^{2} }}{{S_{n + 1} S^{*} I^{*} }} - \frac{\alpha }{{E^{*} }}\left( {p\left( {\frac{{S^{*} }}{{S_{n + 1} }}} \right) + p\left( {\frac{{I_{n + 1} }}{{I^{*} }}} \right) + p\left( {\frac{{E^{*} }}{{E_{n + 1} }}\frac{{I_{n} S_{n + 1} }}{{I^{*} S^{*} }}} \right) - p\left( {\frac{{I_{n} }}{{I^{*} }}} \right)} \right) \\ & \quad - \frac{{\omega I^{*} }}{{S^{*} E^{*} }}\left( {p\left( {\frac{{I_{n + 1} E^{*} }}{{I^{*} E_{n + 1} }}} \right) + p\left( {\frac{{E_{n + 1} I^{*} }}{{E^{*} I_{n + 1} }}} \right)} \right) - \frac{{\alpha R^{*} }}{{S^{*} E^{*} }}\left( {p\left( {\frac{{R_{n + 1} E^{*} }}{{R^{*} E_{n + 1} }}} \right) + p\left( {\frac{{E_{n + 1} R^{*} }}{{E^{*} R_{n + 1} }}} \right)} \right). \\ \end{aligned}$$

Hence, the sequence ${\left\{w(n)\right\}}_{n=1}^{\infty }$ for any $n\ge 0$ is monotonic decreasing. Since $0\le w(n)$ and $\underset{n\to \infty }{\mathrm{lim}}(w\left(n+1\right)-w\left(n\right))=0$, we conclude that $\underset{n\to \infty }{\mathrm{lim}}{S}_{n+1}={S}^{*}$, $\underset{n\to \infty }{\mathrm{lim}}{E}_{n+1}={E}^{*}$, $\underset{n\to \infty }{\mathrm{lim}}{I}_{n+1}={I}^{*}$ and $\underset{n\to \infty }{\mathrm{lim}}{R}_{n+1}={R}^{*}$. This completes the proof.

**Figure 5:**
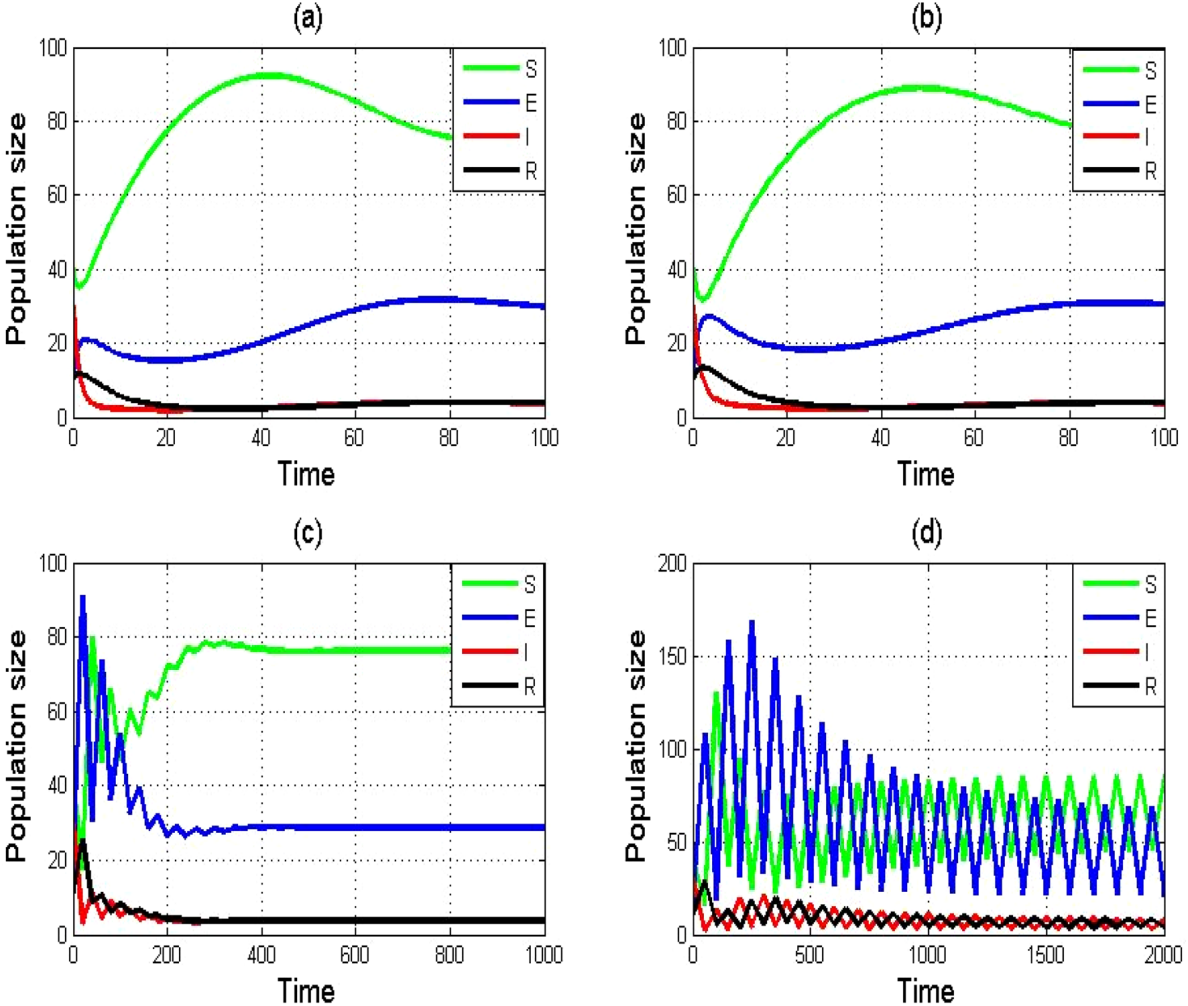
Numerical simulation of SEIR model $\left(1\right)$ by using NSFD scheme with $\left( {\mathbf{a}} \right)\,h = 0.01,\left( {\mathbf{b}} \right)\,h = 1,\left( {\mathbf{c}} \right)\,h = 20,\left( {\mathbf{d}} \right)\,h = 50$. Remaining parameters are set as $\varphi = 4.5,\tau = 0.0{99925},\psi = 0.02,\theta = 0.1503,\alpha = 0.0125,\delta = 0.625,\sigma = 0.125.$

The numerical illustration in Fig. 5a–d additionally illustrates that, for any step size, the NSFD scheme (5) solutions converge to the DEE point if ${R}_{0}>1$. This reveals the NSFD scheme's unconditional convergence.

## Conclusions

In the present paper, a nonlinear epidemic model of a typhoid fever disease is numerically studied using two finite difference schemes. It was shown that the spread of the disease is mostly determined by the rate of contact with sick individuals within a community. The RK-4 and NSFD schemes are employed to discuss the dynamical characteristics of DFE and DEE points, including their local and global stabilities. The findings demonstrate that the NSFD method provides precise numerical solutions while eliminating drawback of RK-4 scheme. The convergence is demonstrated that presents that the NSFD scheme retains their stability and positivity characteristics. The key benefits of NSFD are demonstrated theoretically as well as numerically which reveal that this scheme have good dynamical behavior even for large time step size. At the same time, the RK-4 scheme cannot exactly sustain the fundamental properties of the original continues model and consequently, it can produce numerical solutions which are not quite the same as the solutions of the original model. The NSFD scheme is an easy approach that exhibits how discrete and continuous models act appropriately and produce results that are mathematically accurate. Figures 3, 4, and 5 depicts that the NSFD scheme (5) remains stable for each step size. This demonstrates that for each step size, the NSFD scheme is positive forever and unconditionally convergent. The RK-4 scheme, however, shows convergence only for lower step sizes, as shown in Fig. 3a–d. We can effectively monitor the spread of typhoid fever disease by utilizing the NSFD system. The results provided in this study are advantageous to humanity as well as in the sector of healthcare. Numerical simulations are included in each section to support our theoretical conclusions.

## Data Availability

The data used to support the findings of this study are included within the article.
